# Catarrh and Its Location in the Human Body

**Published:** 1895-02

**Authors:** W. W. Pugh


For Texas Medical Journal.

### CATARRH AND ITS LOCATION IN THE HUMAN BODY.

BY W. W. PUGH, M. D.

IT PERVADES the entire mucous membrane, or more minutely speaking, it affects the mucous lining of the ear, eyes, nose, throat, bronchial tubes, alimentory canal, rectum, vagina and uterus, or womb.

Headache is often caused from catarrh of the frontal sinuses. Sore eyes is caused from catarrh.

Deafness is often caused from catarrh of the middle ear or eustachian tubes.

A discharge from the nose is caused from catarrh of the mucuous lining of the nose or its hidden cavities or sinuses.

Hoarseness is the indication of catarrh of the pharynx, which causes the vocal cords to become enlarged and thickened, which produces the harsh sound while speaking. We often find catarrh of the mucous lining of the tubes in the liver and kidneys.

Female urinary derangements are easily traceable to catarrh of the pelvic organs.

And last, but not least, hemorrhoids, piles and constipation, in a large measure, are caused from catarrh of the mucous membrane of the lower bowel.

Now, as I have realized this to be true, and have treated a great many cases affected with catarrh with good results, I have invented an article which I have called Pugh's Douche Receptacle, for the treatment of catarrh of the rectum, bladder, vagina and womb. It is so made or arranged that any one, the most delicate, can use the proper medicines indicated with the aid of this Receptacle, with the greatest ease and comfort. With the medicated wash in a fountain syringe, the patient can sit upon the Receptacle and introduce the nozzle in rectum or vagina, which is much easier than the old way of sitting over a pan or bowl while taking the douche, which is a great strain on the muscles of lower limbs. I have noticed catarrh often follows the "grippe" as a sequel, just as it does in measles and other diseases affecting the mucous membrane, and in these cases I have not been disappointed in my treatment, and catarrh of this nature is most always certain to affect the nervous system.

Therefore, a good mucous alterative and nerve stimulant, with the cleansing of the affected parts daily, makes a good team to pull your patient out of a distressing condition.

